# Clinical Course of Children with Chronic Suppurative Lung Disease or Bronchiectasis Infected with *Pseudomonas aeruginosa*

**DOI:** 10.3390/children9121822

**Published:** 2022-11-25

**Authors:** Elpiniki Kartsiouni, Stylianos Chatzipanagiotou, Angeliki Galani, Dafni Moriki, Olympia Sardeli, Spyridon Prountzos, Efthymia Alexopoulou, Ioanna Loukou, Kostas N. Priftis, Konstantinos Douros

**Affiliations:** 1 Pediatric Allergy and Respiratory Unit, 3rd Department of Pediatrics, Attikon University Hospital, School of Medicine, National and Kapodistrian University of Athens, 12462 Athens, Greece; 2 Department of Medical Biopathology, Eginition Hospital, School of Medicine, National and Kapodistrian University of Athens, 11528 Athens, Greece; 3 2nd Radiology Department, Attikon University Hospital, National and Kapodistrian University of Athens, 12462 Athens, Greece; 4 Cystic Fibrosis Department, Agia Sofia Children’s Hospital, 11527 Athens, Greece

**Keywords:** *Pseudomonas aeruginosa*, bronchiectasis, chronic suppurative lung disease, children

## Abstract

Children with chronic wet cough and without cystic fibrosis (non-CF) may suffer from chronic suppurative lung disease (CSLD) or bronchiectasis. *Pseudomonas aeruginosa* (Pa) can be one of the offending microbes in these children. The present study aimed to describe the clinical course of children with the above two conditions who were infected with Pa. Data of 54 children with CSLD/bronchiectasis who were diagnosed and attended in our department were retrospectively analysed through a Cox proportional hazard model, with age, presence of bronchiectasis, use of inhaled colistin, azithromycin, inhaled hypertonic saline as the covariates. In 42 of the 54 patients, there was no identifiable cause or underlying chronic disorder. Microbiological clearance was defined as the absence of daily wet cough for four months along with four negative cultures taken during the last four consecutive follow-up visits. Multivariate analysis was performed with a Cox proportional hazard model with time to microbiological clearance as the outcome. Results are described as Hazard Ratios (HR) with 95% Confidence Intervals (95%CI). Nebulised antibiotics and the presence of bronchiectasis were statistically significant predictors of remission (HR: 3.99; 95%CI: 1.12–14.14; *p* = 0.032, and HR: 0.24; 95%CI: 0.08–0.71; *p* = 0.010). In conclusion, the rate of microbiological clearance increases with the use of inhaled colistin and decreases when there is established bronchiectasis.

## 1. Introduction

Chronic endobronchial infections in children not suffering from cystic fibrosis (non-CF) are characterized as chronic suppurative lung disease (CSLD) or bronchiectasis. Most probably, these two conditions do not represent distinct entities but are rather parts of the spectrum of the chronic infection of conducting airways [1,2].

Both CSLD and bronchiectasis are characterized by a chronic, wet and sometimes productive cough, with or without other accompanying features, such as exertional dyspnoea, reactive airway disease, recurrent pulmonary infections, clubbing, and growth failure. Although the clinical picture cannot differentiate between the two conditions, bronchiectasis is also accompanied by abnormal radiographic appearance of the conducting airways with dilation of the bronchial lumen and bronchial wall thickening; such findings are not present in CSLD. Hence, the distinction between these two similar entities is based on chest high-resolution CT scan (HRCT) which shows the aforementioned radiological features in bronchiectasis whereas it is non-diagnostic in CSLD [1,3].

The pathogenesis of both conditions is based on Cole’s vicious cycle hypothesis where an initial bacterial endobronchial infection leads to neutrophilic inflammation and impaired airway clearance that results in airway damage, further growth and spread of the bacteria, and eventually, the establishment of chronic infection [4]. Bronchiectasis represents the latter stage of this pathological process.

*Pseudomonas aeruginosa* (Pa) rarely infects the lung without an underlying immunity defect or impairment of mucociliary clearance. It is a well-known and common pathogen in cystic fibrosis (CF), especially in patients with advanced disease. It has been shown that Pa is associated with an accelerated decline of lung function, deterioration of the radiographic features, increase in the number of exacerbations, and in general, it is considered a significant indicator of the severity of bronchiectasis in both adult and pediatric patients [5,6,7,8,9].

The association of Pa with poor clinical outcomes renders its early detection of great importance in patients with chronic endobronchial infections. The present study aimed to describe the clinical course of non-CF children with CSLD or established bronchiectasis who were infected with Pa.

## 2. Materials and Methods

The present study was conducted in the Pediatric Pulmonology Unit of the Attikon University Hospital in Athens, which is one of the main tertiary referral centres for pediatric pulmonary disorders in Greece. We retrospectively analysed data from 54 non-CF children with CSLD/bronchiectasis, who had been diagnosed and followed in our department during the last 15 years. The criteria for inclusion in the study were the presence of a chronic wet or productive cough and the isolation of Pa at least once in the sputum, cough/throat swabs, or bronchoalveolar lavage (BAL) cultures. For the purposes of this study, we defined microbiological clearance as the absence of wet/productive cough for four months along with three negative cultures taken during the last three consecutive follow-up visits. Thus, we had two groups of patients namely, those with and those without microbiological clearance. There were no exclusion criteria.

Investigations in all patients included sweat test and/or CF gene mutation analysis, and measurement of serum immunoglobulins and IgG subclasses. Nasal nitric oxide test and high-speed video microscopy analysis were performed in patients who apart from the chronic wet cough had at least one of the clinical characteristics of primary ciliary dyskinesia (PCD), namely, organ laterality defect, unexplained neonatal respiratory distress, early onset year-round daily nasal congestion, and recurrent or persistent otitis media.

Data regarding the time of chronic wet/productive cough commencement, the time of referral to our department (which coincided with the time of diagnosis of CSLD/bronchiectasis), the duration of follow-up, and any factors potentially related to the aetiology, were retrieved from the medical records. Microbiological data and the percent-predicted FEV1 (ppFEV1) values (calculated with the National Health and Nutrition Examination Survey III reference equations) of all patients with valid spirometries were also retrieved. The number of exacerbations (as defined elswere [10]) from the time of the first Pa isolation to the time of microbiological remission or the end of follow-up, was determined. All patients received ciprofloxacin per os for at least 3 weeks after the isolation of Pa, and started (if they had not already been on) daily chest physiotherapy. Some of them also received long-term treatment with twice-daily inhaled colistin and/or thrice-weekly azithromycin and/or 7% nebulized hypertonic saline. Three-monthly follow-up visits were arranged for all children. In all visits, sputum or cough/throat swabs cultures were obtained and spirometry was performed in all patients ≥5 years old who were able to cooperate. Exacerbations were usually treated with a per os course of a broad-spectrum antibiotic, mostly amoxicillin/clavulanic acid, if the cultures were negative or did not isolate Pa. If symptoms were not controlled, or Pa was isolated, the treating physician could use a course of ciprofloxacin or admit the child for IV antibiotics.

All chest high-resolution computed tomography (HRCT) scans, which were obtained before or at about the same time as the isolation of Pa, were re-evaluated and scored by two pediatric radiologists (E.A., and S.P.), who were aware of the children’s clinical history but not of the original radiologic diagnosis; any discrepancies among them were resolved with discussion. The criteria for the diagnosis of bronchiectasis with HRCT were dilatation of bronchi with a broncho-arterial ratio > 0.80 [11,12]; parallel bronchial walls in a longitudinal section (tram sign); visualization of bronchi within 1 cm of pleura. The modified Bhalla score was used to quantify the severity of bronchiectasis [13].

Statistical analysis: Variables are described as medians with interquartile range (IQR). Univariate comparisons were performed with Fisher’s exact test and Wilcoxon rank-sum test. Multivariate analysis was performed with a Cox proportional hazard model with time to microbiological clearance as the outcome, and age, presence of bronchiectasis, use of inhaled colistin, azithromycin, and inhaled hypertonic saline, as the covariates. Results were described as Hazard Ratios (HR) with 95% Confidence Intervals (95%CI). In a sensitivity analysis, we excluded patients with underlying chronic disorders. The proportionality assumption was checked with the Schoenfeld test.

## 3. Results

Fifty-four children with CSLD or bronchiectasis, and isolation of Pa in at least one clinical sample, were identified through the clinical records.

Three patients suffered from PCD, three were neurologic patients with swallowing dysfunction and chronic microaspiration, three had tracheoesophageal atresia, one had Crohn disease and was treated with infliximab, one had Job syndrome, and one had anhidrotic ectodermal dysplasia with immunodeficiency. The remaining 42 (77.8%) patients had no identifiable cause. Twenty-four (44.4%) patients were receiving inhaled corticosteroids. The patients’ median (IQR) number of exacerbations during the follow-up period was 6 (2–8). The patients’ characteristics at baseline and during the follow-up period are shown in Table 1. There were only 16 patients who had valid spirometries both at the time of Pa isolation and at the end of the follow-up period with no difference between the ppFEV1 values at the two time points (93.2 ± 1.3, and 94.0 ± 1.5, respectively, *p* = 0.43). The same comparison was also performed in the subgroup of 8 patients who attained remission and no difference was found (93.7 ± 2.0, and 94.4 ± 2.2, respectively, *p* = 0.68).

The Cox proportional hazards model showed that the use of nebulised colistin and the presence of bronchiectasis at HRCT scan were statistically significant predictors of microbiological clearance (HR: 3.99; 95%CI: 1.12–14.14; *p* = 0.032, and HR: 0.24; 95%CI: 0.08–0.71; *p* = 0.010). The estimated HRs indicated that patients on colistin were, on average, approximately four times more likely to achieve remission compared to patients not treated with this drug whereas patients with radiologically confirmed bronchiectasis were, on average, approximately four times less likely to achieve remission compared with patients without bronchiectasis. Age, use of azithromycin, and hypertonic saline inhalation were also included as covariates in the Cox model but no significant correlation between them and microbiological clearance was established. In a sensitivity analysis, we excluded patients with diagnosed underlying chronic disorders (n = 12) and repeated the analysis; the observed relationships were similar to those of the main analysis. Details on the results from the main and the sensitivity analysis models are provided in Table 2.

The Schoenfeld tests showed that there was no violation of the proportional assumption in our model (all *p* > 0.05).

## 4. Discussion

The absence of clear national or international guidelines for the eradication and suppression of Pa in children with non-CF chronic endobronchial infection resulted in a non-unanimous approach from the doctors serving in our department, with treatment decisions being based on the subjective clinical judgment of each physician. Nevertheless, the different therapeutic attitudes were an advantage for this retrospective study which aimed to explore the efficacy of the various treatment approaches. The lack of a credible definition of remission made us invent the functional term of microbiological clearance and use it as the endpoint of the study.

The study showed that treatment with inhaled colistin increased whereas the presence of radiologically confirmed bronchiectasis reduced the rate of microbiological clearance. Sensitivity analysis with the exclusion of patients with diagnosed underlying chronic disorders did not change substantially our results. Our data were unable to show any significant correlation between microbiological clearance and the two other treatment modalities namely, azithromycin and inhaled hypertonic saline.

The presence of Pa in the airways of patients with non-CF bronchiectasis has been associated with accelerated lung function decline and increased morbidity and mortality [7,14,15]. However, despite the advantages in our knowledge of Pa bronchial infections, much of our understanding is still extrapolated from CF [16]. Furthermore, it is difficult to estimate the true impact of primary Pa infection in pediatric patients. Indeed, specific data on children are lacking and most of the available studies in adults have concentrated on chronic—and not primary—infection.

There is no universally accepted definition of chronic Pa infection with most of the currently used definitions being based on microbiological results from sputum cultures in CF patients [17]. However, young children rarely expectorate sputum and this fact dictates the use of alternative methods for microbiological sample collection. BAL is taken through an interventional procedure and cannot be used in the usual follow-up. Practically, cough and throat swabs are the only possible alternatives to sputum. These latter methods, however, despite being very convenient, place considerable uncertainty on the results as they lack the sensitivity of sputum or BAL cultures [18]. Furthermore, in cases of biofilm formation, it can be difficult to recover all the clinically significant bacteria through conventional cultures [19]. For the above reasons, the isolation of Pa for the first time cannot exclude the existence of a chronic Pa infection, especially if the symptoms persist for a long period and/or the child has repeatedly not responded to conventional antibiotics. The difficulty and ambiguity in characterizing a first-time Pa isolation as new onset or chronic infection can justify the use of inhaled antibiotics for prolonged periods.

Inhaled antibiotics attain very high drug concentrations in the airways without the adverse effects that are observed when they are administered through systemic routes [20,21]. Although many inhaled antibiotics have been developed and are currently in use, only colistin was available for our patients since it is the only inhaled antibiotic that is compensated by the national health insurance system for non-CF patients. Long-term treatment with inhaled antibiotics reduces the number of exacerbations, decreases bacterial load, and improves pulmonary function in several chronic endobronchial infections [20,22,23,24]. Our data are in general agreement with the above results as they demonstrated that the prolonged use of inhaled colistin increased the rate of microbiological clearance.

Bronchiectasis is the end stage of chronic endobronchial infections [2] and its presence is correlated with the severity of clinical symptoms and the intensity of neutrophilic inflammation in the airways [3]. As such it is inherently hard—though not impossible—to remit [11,25]. Given so, our finding that the presence of bronchiectasis reduced the rate of microbiological clearance was somehow expected as bronchiectasis denotes the most severely affected patients.

The large percentage of patients treated with inhaled steroids (ICS) should be attributed to the lack of a simple test for the diagnosis of asthma. Because of this physicians prescribe ICS in a seemingly arbitrary way. Quite often the diagnosis of asthma is based on a therapeutic trial of ICS and an assessment of the child’s response. Nevertheless, this approach is not always helpful in children with bronchiectasis because of the confounding effect of co-administered drugs [26].

The present study suffers from some limitations. First of all, it was a single-centre retrospective observational study that reflected the population served by our department, and the results cannot be generalized. The number of patients was relatively small and so type 2 errors (false-negative results) concerning treatment with azithromycin and nebulized hypertonic saline, may have occurred. Finally, we used microbiological clearance as the end-point of our study which is quite different from true clinical remission.

## 5. Conclusions

Inhaled colistin is a useful therapeutic modality in children with CSLD and non-CF bronchiectasis. Children with CSLD have a more favourable clinical course compared to children with established bronchiectasis.

## Figures and Tables

**Table 1:** Patients’ characteristics at baseline and during the follow-up period.

	Patients with Microbiological Clearance (n = 22)	Patients without Microbiological Clearance (n = 32)	*p*
Gender (M/F)	9/13	22/10	0.06
Positive SPT or RAST *	7	9	0.77
History of wheezing	8	11	0.88
Inhaled steroids	10	14	0.90
Number of patients with at least one IV antibiotic course	1	2	0.80
Median (IQR) number of exacerbations	5 (2–7)	6 (3–8)	0.71
Median (IQR) age of wet cough onset (years)	1.3 (0.4–4.0)	1.1 (0.5–2.3)	0.57
Median (IQR) duration of wet cough before the isolation of Pa (years)	1.5 (0.9–3.1)	1.5 (1.1–2.7)	0.88
Median (IQR) duration of follow-up (years)	1.9 (1.0–3.6)	1.8 (1.1–3.1)	0.88
Median (IQR) age of first Pa isolation (years)	2.6 (1.5–6.1)	2.6 (1.5–4.5)	0.58
Patients with Pa isolation more than once	1 (4.5%)	9 (28.1%)	0.036
Other isolated bacteria		
- Pneumococcus - Haemophilus influenzae - Moraxella catarrhalis - Staphylococcus aureus - Gram-negative	2 (9.0%) 5 (22.7%) 3 (13.6%) 3 (13.6%) 5 (22.7%)	4 (12.5%) 3 (9.3%) 4 (12.5%) 3 (9.3%) 10 (31.2%)	0.69 0.25 0.90 0.67 0.55
Children on long-term inhaled colistin (n; %)	18 (81.8%)	11 (34.4%)	0.001
Median (IQR) duration of treatment with inhaled colistin (months)	1.2 (0.8–2.7)	1.4 (1.0–3.1)	0.82
Children on long-term azithromycin (n;%)	15 (68.1%)	14 (43.7%)	0.08
Median (IQR) duration of treatment with azithromycin (months)	0.8 (0.5–1.7)	1.1 (0.8–2.3)	0.52
Children on long-term nebulized hypertonic saline (n;%)	17 (77.2%)	17 (53.1%)	0.09
ppFEV1 (IQR) **	93 (89.5–98.5)	90.5 (89–96.5)	0.71
Children with radiologically confirmed bronchiectasis	6 (27.2%)	20 (62.5%)	0.011
Median (IQR) modified Bhalla score ***	3 (2–4)	4 (2–5)	0.35

Pa: *Pseudomonas aeruginosa*; IQR: Interquartile range; Gram-negative: Pathogenic Gram-negative bacteria (Enterobacteriaceae, Achromobacter xylosoxidans, Stenotrophomonas maltophilia); * Positive SPT or RAST: Positive skin prick tests or significant levels of specific IgE antibodies to common aeroallergens; ** Percent predicted FEV1 (ppFEV1) was measured only in 16 patients; *** Measured only in 26 patients with established bronchiectasis.

**Table 2:** Results from the multivariate analysis with a Cox proportional hazard model for both the main analysis (n = 54), and sensitivity analysis (n = 42) with the 12 patients with diagnosed underlying chronic disorders having been excluded.

	Main Analysis			Sensitivity Analysis (with 12 Patients Excluded)		
	HR	95%CI	*p*	HR	95%CI	*p*
Age	1.13	0.99–1.28	0.06	1.13	0.97–1.32	0.11
Bronchiectasis	0.24	0.08–0.71	0.010	0.18	0.05–0.64	0.008
Inhaled colistin	3.99	1.12–14.14	0.032	5.47	1.02–29.37	0.047
Azithromycin	1.34	0.46–3.93	0.58	1.61	0.46–5.59	0.44
Inhaled hypertonic saline	0.80	0.22–2.87	0.74	0.51	0.12–2.15	0.36

HR: Hazards Ratio; 95%CI: 95% Confidence Interval.

## Data Availability

The data presented in this study are available on request from the corresponding author. The data are not publicly available due to privacy and associated regulatory constraints.
